# Long-term survival of the cemented Müller CDH stem: a minimum follow-up of 10 years

**DOI:** 10.1007/s00402-018-3009-7

**Published:** 2018-07-25

**Authors:** Yves Salentiny, Lukas Zwicky, Peter E. Ochsner, Martin Clauss

**Affiliations:** Clinic for Orthopedics and Trauma Surgery, Kantonsspital Baselland, Rheinstrasse 26, 4410 Liestal, Switzerland

**Keywords:** Aseptic loosening, CDH, Cemented stem, Hip dysplasia, Risk factors, Total hip arthroplasty

## Abstract

**Introduction:**

Total hip arthroplasty in patients with altered anatomy of the hip and femur, such as in congenital dysplasia of the hip, is challenging and often requires specially designed stems. Müller straight stems have shown excellent long-term results; however, long-term data on the analogous cemented Müller CDH stem are still missing. The aim of this study was to analyze long-term survival, identify potential risk factors for aseptic loosening, and analyze radiological outcome of the cemented Müller CDH stems.

**Materials and methods:**

Between 01/1985 and 06/2005, 95 Müller CDH stems (Zimmer, Winterthur, Switzerland) made up of 3 different materials were cemented using 2 different bone cements: 38 of stainless steel/high-viscosity cement, 31 of a cobalt-chrome-based alloy (CoCr)/low-viscosity cement, and 26 of a titanium-based alloy (Ti)/low-viscosity cement. All patients had a prospective clinical and radiological follow-up according to the standards of our institution. The cumulative incidence for revision of the stem was calculated using a competing risk model. To identify demographic and implant-related risk factors for aseptic loosening of the stem, a multivariate regression model for competing risks was performed.

**Results:**

The cumulative risk of revision at 15 years was 12.5% (95% CI 6.6–20.5%) for aseptic loosening of the stem as endpoint, with marked differences for the various stem materials used: stainless steel 2.7% (0.2–12.3%), CoCr 12.9% (4.0–27.3%), and Ti 24.5% (9.6–43.1%). Regression modeling revealed that Ti stems in combination with low-viscosity cement (HR 10.2) and implantation with an axis deviation greater than 3° (HR 3.8) are risk factors for aseptic loosening.

**Conclusions:**

Long-term survival of the cemented Müller CDH stem is comparable to other Müller-type straight stems and uncemented implants. Similar to the original Ti Müller straight stem, the Ti Müller CDH stem also showed an increased risk for aseptic loosening and should, therefore, no longer be used.

## Introduction

Treating osteoarthritis of the hip in patients with an altered anatomy is technically more challenging when using standard implants. Such an altered anatomy is observed in patients with congenital dyplasia of the hip (CDH), in patients with a severe varus position of the femoral neck, an increased antetorsion or a narrow medullary canal. Two-dimensional [1, 2] and three-dimensional analyses in an Asian [3, 4] and Caucasian [5] CDH population further demonstrated a straight femur with a distalized anterior femoral bow, making it even more difficult to fit a standard implant in the femoral canal in these particular cases.

Both Charnley and Harris developed special cemented CDH stems to address the difficulties faced in this specific patient group. Recently, long-term results were published showing a 20-year survival rate with aseptic loosening as the endpoint in 63% of the Charnley and in 78% of the Harris CDH stem [6]. Excellent results have been reported for the small variant of the Exeter stem for patient with small femurs [7]. Good long-term survival rates in CDH patients have also been reported for uncemented [8] and modular stem designs [9] with survival rates of 96% after 12 years and 97% after 8-year follow-up, respectively.

In hip arthroplasty, the original Müller straight stem is one of the most common implanted cemented straight stems with excellent long-term results [10–13]. The cemented Müller CDH stem belongs to the same implant series. Appropriate indications for its usage amongst others are hip joints with small and hypoplastic bony anatomy with narrow femoral canals as well as osteoarthritis secondary to congenital or childhood disorders of the hip. However, up to now, no data are available showing its long-term outcome.

The aim of this study was to analyze long-term survival, identify potential risk factors for aseptic loosening, and analyze radiological outcome of the cemented Müller CDH stems.

## Materials and methods

### Demographics

Between 01/1985 and 06/2005, a consecutive series of 95 total hip arthroplasties (THA) using a Müller CDH straight stem (Zimmer, Winterthur, Switzerland), were performed in 86 patients with bony abnormalities. All patients were followed prospectively with a standardized follow-up protocol. All surgeries were carried out personally or under direct supervision of one of the authors (PEOXXX).

75 of the 86 patients were female. The median age at the time of surgery was 65 years (range 43–88 years). Indications for THA were primary osteoarthrosis (OA) in 50 cases, CDH in 24 cases, fracture and avascular necrosis in 6 cases respectively, rheumatoid OA in 5 cases, and OA secondary to Legg–Calvé–Perthes disease in 4 cases. All patients were operated in supine position through a lateral transgluteal approach. Indications to use the Müller CDH straight stem, instead of a regular stem, were (1) a narrow medullary canal and/or (2) a deteriorated proximal femoral geometry, e.g., lacking of a femoral calcar and/or (3) cases of dysplasia and malpositioned greater trochanter additionally needing a trochanteric osteotomy [14]. During the whole-study period, operative technique (except the type of bone cement) and patient aftercare remained unchanged.

### Implants

**Fig. 1:**
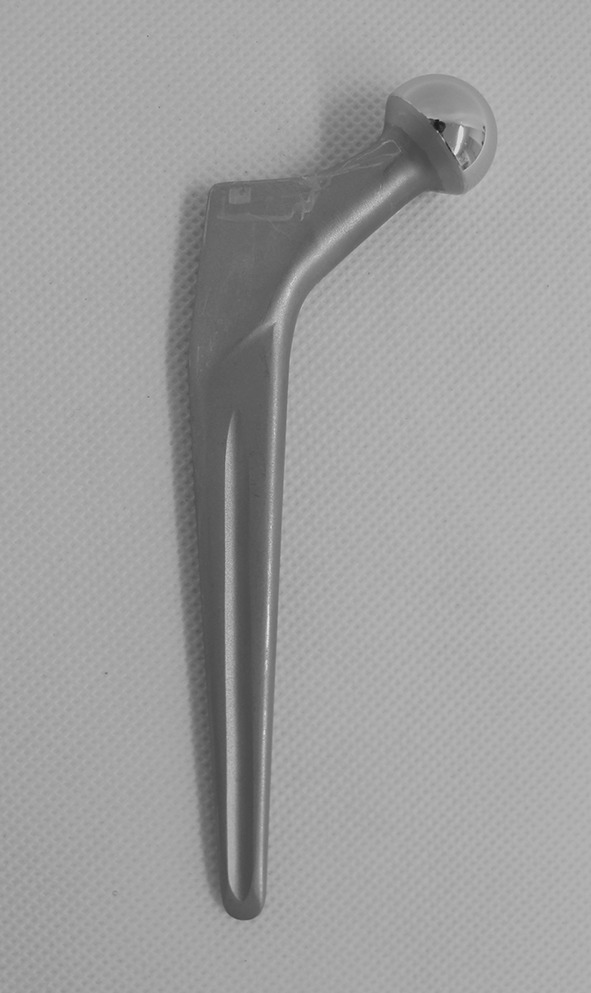
Müller CDH stem (Zimmer, Winterthur, Switzerland). The stem is smooth blasted. The anterior and posterior sides present a longitudinal groove

The Müller CDH straight stem is a double-tapered design and was manufactured as a monobloc stem with a 22-mm head and a CCD angle of 130° (Fig. 1). It was available in five different sizes. Although the manufacturer did not change the design of the stem over the years, it was manufactured in three different biomaterials, which were implanted in our institution: 31 CoNiCrMo (CoCr) stems (1985–1997), 26 Ti-6Al-7Nb (Ti) stems (1988–1993), and 38 stainless steel stems (1997–2005, Table 1).

**Table 1:** Characteristics of the different implanted Müller CDH straight stems

*N*	31	26	38
Material	CoNiCr	Ti6Al7Nb	Stainless steel
Surface roughness (R_a_)	1.0 ± 0.5 µm		
Cement	Sulfix-60	Sulfix-60	Palacos R

All stems were cemented using the second-generation cementing techniques with an autologous distal bone plug. Two different types of antibiotic-free cement were used: low-viscosity cement (Sulfix-60, Zimmer) between 1985 and 1997 (57 hips, CoCr and Ti stems), and high-viscosity cement (Palacos R, Heraeus, Wehrheim, Germany) between 1998 and 2005 (38 hips, stainless steel stems). In 17 hips, a trochanteric osteotomy was performed to allow proper medullary canal entry. The stem was combined with two different cup systems, namely an acetabular reinforcement ring (ARR, Zimmer) with a cemented polyethylene cup (67 hips) or an uncemented pressfit cup (SL-II, Zimmer, 28 hips).

### Follow-up and radiological analysis

Patients were followed prospectively according to the standardized protocol at our institution [13]. The follow-ups were scheduled at 4 months, 1, 2, and 5 years, and every 5 years thereafter.

**Fig. 2:**
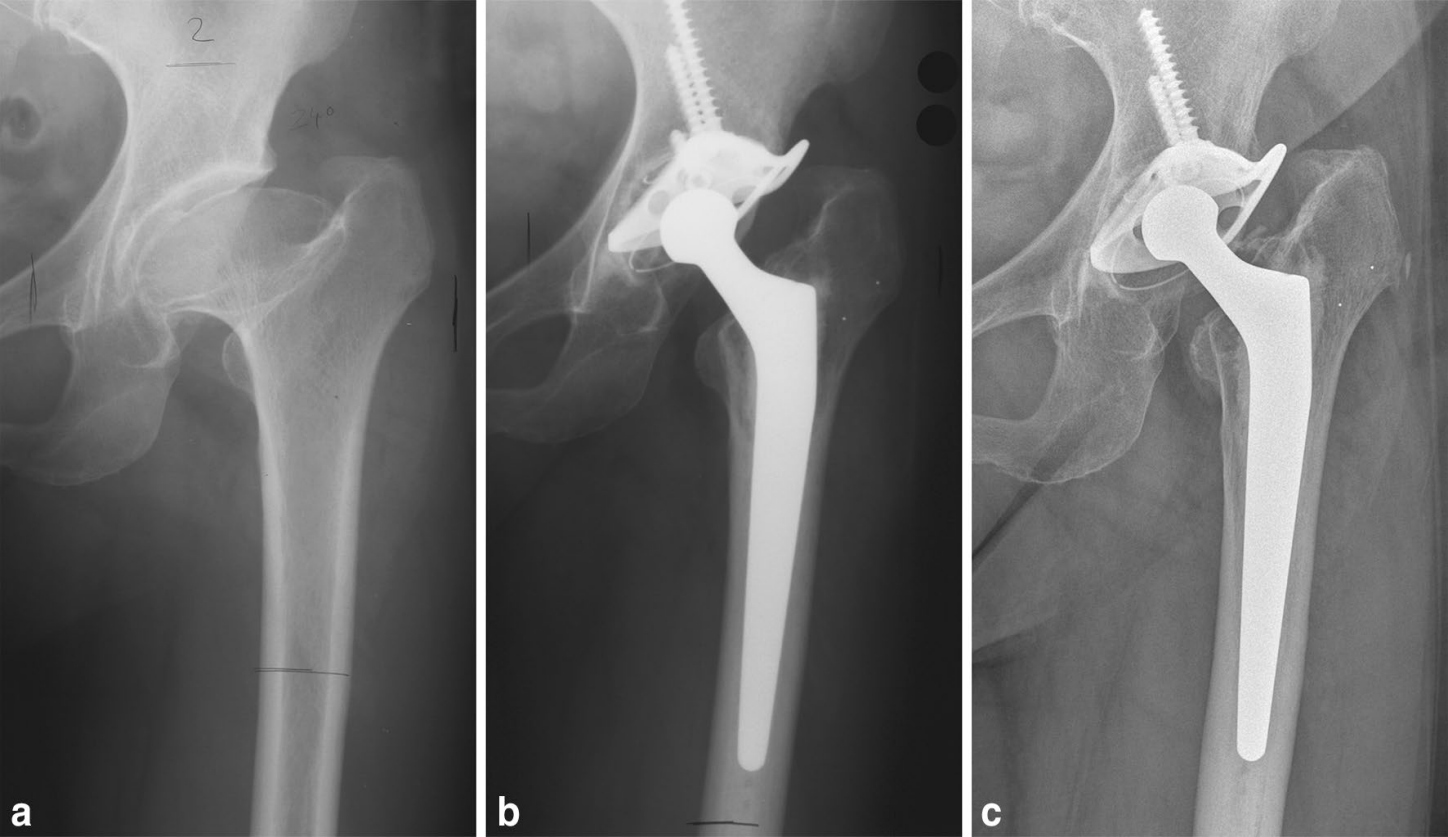
**a** 55-year-old female patient with a Perthes hip, **b** reconstruction with an acetabular reinforcement ring and CDH stem, **c** at 18-year follow-up, with no signs of any radiological changes

Standardized anterior–posterior radiographs centered on the symphysis, showing that the entire prostheses were performed at follow-ups. The radiological assessment was based on the most recent radiograph available from the follow-ups or in case of a revision of the stem, the last available radiograph prior to revision surgery [13, 15] (Fig. 2).

Osteolysis at the bone–cement interface was defined as any progressive and newly developed endosteal bone loss with a diameter > 3 mm [13, 16]. A radiolucent line at the prosthesis–cement interface, not visible on the first postoperative radiograph, was considered debonding of the stem–cement interface [13]. The alignment of the stem was evaluated on the radiograph 4 months postoperatively by comparing the femoral bone axis to the stem axis. The axis of the femoral bone was determined by drawing a line through the center of two lines, drawn at two different heights of the femur, at the level of the prosthesis, perpendicular to the cortex. An analogous axis was drawn through the stem of the prosthesis. A varus or valgus alignment of less than 3° was considered to be neutral [17]. Subsidence was defined as being an increase of radiolucency between the shoulder of the prosthesis and proximal cement in Gruen zone 1 [12]. Cortical atrophy of the femur was defined as being a longitudinal thinning of the femoral cortex due to intracortical porosis, without any measurable changes of the diameter of the femur [18]. Debonding, osteolysis, and cortical atrophy of the femur were evaluated in the Gruen zones [19]. Stems were considered radiographically loose when osteolysis in all Gruen zones was present and/or subsidence was > 10 mm [15, 20].

Ambiguous findings were discussed with the senior investigator (**blinded**) and agreed upon.

### Statistics

A survival analysis with death as a competing risk was performed to determine the cumulative revision rate (CRR) of: (1) aseptic loosening of the stem and (2) stem loosening for any reason. Patients without any revision were censored at the date of last contact. The analysis was halted once the number of patients at risk was smaller than 25.

In addition, time to aseptic loosening of the stem was analyzed using a multivariate proportional hazards model for the subdistribution of death as a competing risk [21]. Factors included in the full model were known risk factors for aseptic loosening of the original Müller straight stem such as age (< 60, 60–70, > 70 years), sex, stem material (CoCr, Ti, and stainless steel), cup fixation (cemented and uncemented), stem size (5.0, 7.5, 10.0, and ≥ 12.5) [15], type of cement (Sulfix, Palacos) [15], and stem alignment (neutral, >|3°|). Selection of the parameter in the final model was performed using the Bayesian information criteria (BIC) [22, 23].

Categorical data were compared using Fisher’s exact test or Pearson’s Chi-square test, while, for continuous variables, a *t* test was used. The level for statistical significance was set at *p* < 0.05.

Statistical analyses were performed using R statistical package version 3.1.3 [24].

## Results

### Follow-up

Twenty-two patients [34 hips, 15 CoCr (48%), 14 Ti (54%), and 5 stainless steel (13%)] had died of causes unrelated to the THA 11 (0.7–23) years after implantation; one patient (stainless steel group) was lost to follow-up after 6 years. The median clinical follow-up was 12 (0.7–30) years.

### Cumulative revision rate

17 stems were revised: 13 for aseptic loosening, 2 due to infection, 1 after recurrent dislocations, and 1 after a periprosthetic femoral fracture. There was one additional isolated cup revision.

The cumulative revision rate (CRR) for aseptic loosening at 15 years was 12.5% (95% CI 6.6–20.5%); the CRR for stem loosening for any reason at 15 years was 16.4% (9.6–24.7%). In contrast, the competing risk for death at 15 years was 32.1% (22.0–42.7%). CRR for aseptic loosening of the stem of the various materials were 2.7% (0.2–12.3%), 12.9% (4.0–27.3%), and 24.5% (9.6–43.1%) for stainless steel, CoCr, and Ti, respectively (*p* = 0.10, Fig. 4).

### Risk factors for aseptic loosening of the stem

The hazard ratios (HR) of the full model are presented in Table 2. Based on these estimates, a model was built using the BIC difference to the null model using a “forward” approach. As Palacos was only used in combination with implants made of stainless steel, the factor cement was merged with the factor material. The final model included stem material/type of cement [CoCr/Sulfix, HR 4.42 (0.54–36.0), *p* = 0.16; Ti/Sulfix, HR 10.2 (1.16–89.1), *p* = 0.04] and stem alignment [HR 3.84 (1.23–12.0), *p* = 0.02].

**Table 2:** Hazard ratios (HRs) for aseptic loosening of the CDH Müller straight stem

Parameter	No failure	Aseptic loosening	Full model HR (95% CI)	*p*	Final model HR (95%CI)	*p*
Age						
<60	28	5	Ref			
60–70	29	6	0.70 (0.18–2.73)	0.60		
>70	25	2	0.48 (0.07–3.36)	0.46		
Sex						
Male	8	3	Ref			
Female	74	10	0.29 (0.03–2.84)	0.29		
Cup fixation						
ARR	58	9	Ref			
SL-II	24	4	3.52 (0.57–21.6)	0.17		
Stem material/cement						
Steel/palacos	37	1	Ref		Ref	
Ti/sulfix	20	6	17.5 (3.11–97.8)	0.001	10.2 (1.16–89.1)	0.04
CoCr/sulfix	25	6	5.15 (0.64–41.5)	0.16	4.42 (0.5–36.0)	0.16
Alignment						
Neutral	60	8	Ref		Ref	
>|3°|	9	5	4.13 (0.85–20.13)	0.08	3.84 (1.23–12.0)	0.02

### Radiological results

For a total of 13 hips, no radiographs were available, leaving 82 hips for radiological analysis. The median radiological follow-up was 10 (0.2–30) years.

Osteolysis was found in 20 of the 82 stems. It appeared primarily on the proximal medial stem side in the Gruen zones 6 (*n* = 14) and 7 (*n* = 13). In 12 of 13 stems revised due to aseptic loosening, osteolysis was present. Sex (*p* = 0.45) and age (*p* = 0.70) had no influence on the occurrence of osteolysis. No stem was rated radiologically loose without being revised.

Debonding was seen in 20 of 82 stems, mainly on the proximal lateral side of the stem in Gruen zone 1. 11 of the 13 stems revised for aseptic loosening showed debonding, while only 9 of the remaining 69 stems not revised for aseptic loosening showed debonding (*p* < 0.001).

A total of 68 stems had neutral alignment. 13 stems were implanted with a varus alignment (3.1–5.2°), and 1 stem had a valgus alignment of 3.5°.

16 of 82 stems showed subsidence with a median of 3 (1–15) mm. 6 of the 13 stems revised for aseptic loosening had subsided of which 5 subsided more than 2 mm (6, 7, 10, 13, and 15 mm).

Cortical atrophy was seen in 35 of 82 cases, mainly in Gruen zones 2 (*n* = 29) and 6 (*n* = 32). Cortical atrophy was seen in 2 of the cases in which the stems were revised for aseptic loosening compared to 33 in the group which were not revised for aseptic loosening (*p* = 0.06). Hips showing cortical atrophy had a statistically significant longer radiological follow-up time [12.7 (6.1) vs. 8.3 (5.5) years, *p* = 0.001].

A trochanteric osteotomy was performed in 17 hips with evident pseudarthrosis in seven cases (Table 3).

**Table 3:** Radiological results

	CoCr	Ti	Stainless steel
No radiological follow-up	3	6	3
Osteolysis	6	7	7
Debonding	7	7	6
Cortical atrophy	13	8	14
Alignment			
Neutral	21	17	30
Varus	6	3	4
Valgus	1	0	0
Subsidence	4	4	8

## Discussion

**Fig. 3:**
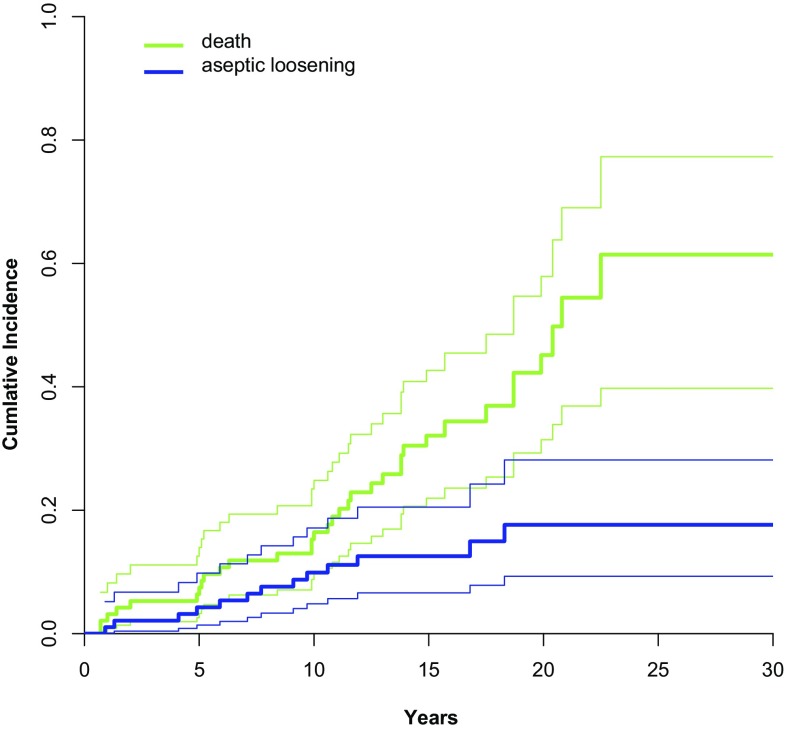
Cumulative incidence (with 95% confidence intervals) for aseptic loosening of the stem and death

The Müller CDH stem shows good long-term survival rates with a cumulative revision rate (CRR) of 12.5% (6.6–20.5%) at 15 years with aseptic loosening as the endpoint (Fig. 3). This is superior to other cemented dysplasia stems [6] and comparable to uncemented dysplasia stems [6, 8, 9, 14, 25–27]. Comparable results have also been published with the small versions of the polished cemented Exeter stem [7].

**Fig. 4:**
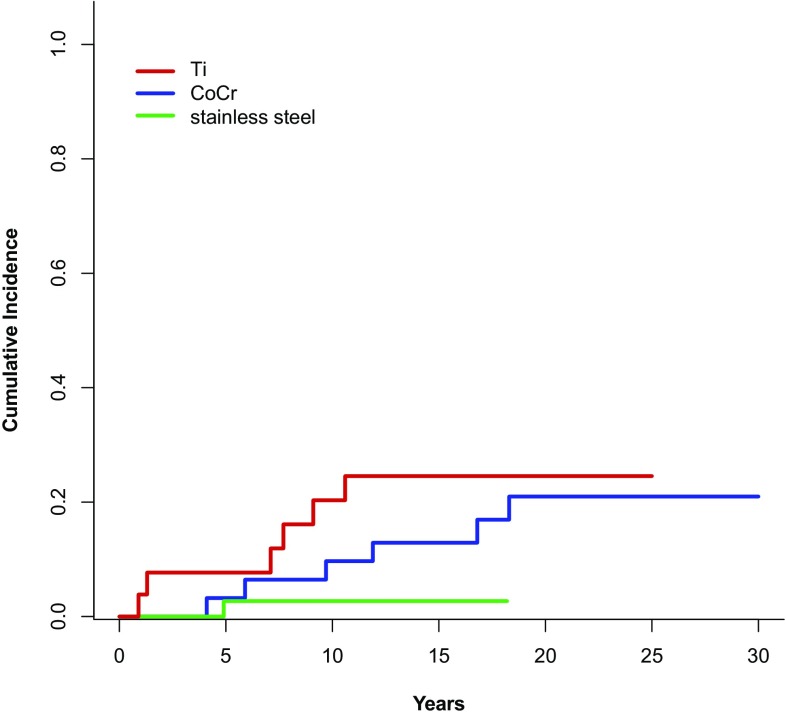
Cumulative risk for revision due to aseptic loosening of the stem: 38 stainless steel (green), 31 CoCr (blue), and 26 Ti (red)

CRR with aseptic loosening as the endpoint was lower for stems made of stainless steel (2.7%) compared to Ti (24.5%) (Fig. 4). The performance of the stainless steel Mueller CDH stem in combination with low-viscosity cement was superior to other cemented stems and similar to uncemented stems [8, 9]. This can be attributed to the higher modulus of elasticity of stainless steel causing less cement damage [28, 29]. Clauss et al. [30] concluded, in their publication, that cemented straight stems should be made of a material with high flexural strength (e.g., steel or CoCr), which seems to be true also for the Müller CDH stem. Analyzing the stems with a high flexural strength, the HR for aseptic loosening of CoCr/sulfix stems was 4.42 (*p* = 0.16) compared to steel/palacos stems. The use of a different type of cement may explain why the CoCr stems showed a tendency toward higher CRR in comparison to stainless steel stems, even though they have approximately equal stiffness. A recently published prospective randomized trial including 711 Muller type straight stems made of CoCr found a statistically non-significant difference in favour for high-viscosity Palacos over low-viscosity sulfix [15].

The Müller CDH stem was developed for hips with altered anatomy showing small femora, or dysplastic hips presenting an increased CCD angle with a small head, allowing for a wedge-type stem with additional cement fixation. It follows the same cementing philosophy as the original Müller straight stem, however, with extended possible indications [18, 31]. For cemented Müller straight stems, Clauss et al. [13] have shown osteolysis to occur mainly in the Gruen zones 6 and 7 with debonding first appearing superolaterally, which is in accordance to the radiological findings of the Müller CDH straight stem. Both stems have a similar double-tapered design with a groove running down the lower two-thirds of the stem allowing for analogous fixation in the femoral shaft. In our series, osteolysis was found in 92% of stems revised for aseptic loosening. Only two of the stems revised showed cortical atrophy. Patients showing cortical atrophy had a longer follow-up period, substantiating the findings of Poss et al. [32] and being in accordance with the original Müller straight stem [13]. Cortical atrophy, therefore, seems to be a rather positive sign.

This study has a few limitations, namely that two different cement types were used and that different cup designs were combined with the Müller CDH stem. It is well known from the Norwegian Arthroplasty register that low-viscosity bone cement is a risk factor for increased aseptic loosening [33, 34]. The authors even concluded that the effect of the cement on aseptic loosening itself might be higher than the prosthesis brand [35]. However, we are not able to prove statistically whether cement or stem material is the more important risk factor for aseptic loosening for stems with a comparable flexural stiffness (CoCr/sulfix vs SS/palacos). As, to the latter, a superior cup may protect an inferior stem which also applies the other way around linking aseptic loosening of the cup to the stem [30, 36]. In our series, 2/3 of the Müller CDH stems were combined with an ARR. This cup has shown to have excellent long-term results in a series of 123 consecutive total hip arthroplasties between 1981 and 1986 [37] as well as in a high number of complex cases between 1984 and 2003 [38]. Another limitation was the use of different stem materials at different time intervals. Although the surgical technique concerning surgical experience and patient care over time remained the same during the whole-study period, the possibility of general improvement in patient care over time cannot completely be excluded.

To our knowledge, the present study is the first one reporting long-term survival and radiological outcome of the cemented Müller CDH stem after a mean of 15 years. The outcome of the stainless steel Mueller CDH stem with low-viscosity cement is similar to other stems with similar indications.

In conclusion, the CDH Müller straight stem shows good-to-excellent long-term survival and substantiates the findings of other CDH implants and Müller straight stems. Similar to the results of the original Müller straight stem, these data suggest that the material of the stem should be of high modulus of elasticity (e.g., stainless steel or cobalt chrome). Cemented CDH Müller straight stems made of Ti should no longer be used and the use of high-viscosity cement is recommended. Therefore, the ongoing use of the Müller CDH stem in contemporary THA for patients which meet the indication criteria would be justified, especially when using modern third-generation cementing techniques.
